# Violapyrones H and I, New Cytotoxic Compounds Isolated from *Streptomyces* sp. Associated with the Marine Starfish *Acanthaster planci*

**DOI:** 10.3390/md12063283

**Published:** 2014-05-30

**Authors:** Hee Jae Shin, Hwa-Sun Lee, Jong Seok Lee, Junho Shin, Min Ah Lee, Hyi-Seung Lee, Yeon-Ju Lee, Jieun Yun, Jong Soon Kang

**Affiliations:** 1 Marine Natural Products Chemistry Laboratory, Korea Institute of Ocean Science and Technology, 787 Haeanro, Ansan 426-744, Korea; E-Mails: hwasunlee@kiost.ac (H.-S.L.); jslee@kiost.ac (J.S.L.); shinjh@kiost.ac (J.S.); yimina82@kiost.ac (M.A.L.); hslee@kiost.ac (H.-S.L.); yjlee@kiost.ac (Y.-J.L.); 2 Department of Marine Biotechnology, University of Science and Technology, 217 Gajungro, Daejeon 305-350, Korea; 3 Bio-Evaluation Center, Korea Research Institute of Bioscience and Biotechnology, 30 Yeongudanjiro, Cheongwon 323-883, Korea; E-Mails: jyun@kribb.re.kr (J.Y.); kanjon@kribb.re.kr (J.S.K.)

**Keywords:** *Streptomyces* sp., violapyrones, anti-cancer activity, α-pyrones, starfish

## Abstract

Two new α-pyrone derivatives, violapyrones H (**1**) and I (**2**), along with known violapyrones B (**3**) and C (**4**) were isolated from the fermentation broth of a marine actinomycete *Streptomyces* sp. The strain was derived from a crown-of-thorns starfish, *Acanthaster planci*, collected from Chuuk, Federated States of Micronesia. The structures of violapyrones were elucidated by the analysis of 1D and 2D NMR and HR-ESIMS data. Violapyrones (**1**–**4**) exhibited cytotoxicity against 10 human cancer cell lines with GI_50_ values of 1.10–26.12 μg/mL when tested using sulforhodamine B (SRB) assay. This is the first report on the cytotoxicity of violapyrones against cancer cell lines and the absolute configuration of violapyrone C.

## 1. Introduction

Marine actinomycetes, isolated from the surface of marine algae and invertebrates, have received increased attention as a potential source because they produce a variety of new bioactive secondary metabolites compared to terrestrial microorganisms [1,2]. As a part of our ongoing research for the discovery of bioactive metabolites from marine bacteria, we isolated a marine actinomycete *Streptomyces* sp. 112CH148 from a crown-of-thorns starfish, *Acanthaster planci*. *A. planci* has a long history in the scientific literature but only few studies have been done on its microbial symbionts [3,4,5]. We tried to isolate bioactive strains from the starfish and found that among the isolates, the strain 112CH148 produces unusual 3,4,6-trisubstituted α-pyrone derivatives.

**Figure 1:**
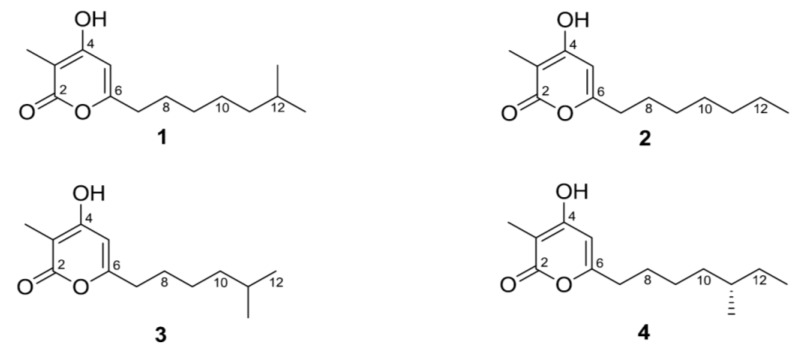
Structures of violapyrones H (**1**), I (**2**), B (**3**) and C (**4**).

α-Pyrones are an important class of lactones having a broad spectrum of biological activities, such as potent anticancer [6], antimicrobial [7,8], antifungal [9,10], antioxidant [11,12,13], androgen like [14], HIV-1 protease inhibitory [15,16] and pheromonal effects [17]. Here, we report the isolation, structure determination of the new 3,4,6-trisubstituted α-pyrone derivatives, violapyrones H (**1**) and I (**2**), and the cytotoxicity of violapyrones (**1**–**4**) (Figure 1).

## 2. Results and Discussion

### 2.1. Isolation of Compounds

The bacterial strain 112CH148 was isolated from the crown-of-thorns starfish, *Acanthaster planci*, collected from Chuuk, Federated States of Micronesia and identified as *Streptomyces* sp. by 16S rRNA sequencing. The strain was cultured in Bennett’s medium (salinity 32 g/L, pH 7.02 before sterilization) at 28 °C for 7 days. Then, the fermentation broth was extracted with EtOAc. Thereafter, two new violapyrones (**1**,**2**) and two known violapyrones (**3**,**4**) were isolated from the EtOAc extract by stepwise gradient open column chromatography followed by reversed-phase HPLC separations.

### 2.2. Structure Determination

**Figure 2:**
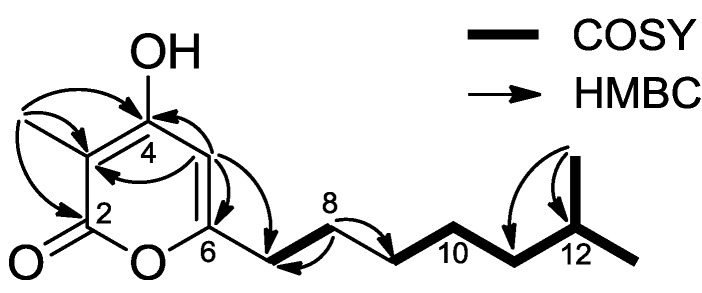
Key HMBC and COSY correlations of **1** in CD_3_OD.

Violapyrone H (**1**) was isolated as a yellowish, amorphous solid. The molecular formula C_14_H_22_O_3_ was deduced from the [M + Na]^+^ peak at *m/z* 261.1466 (calcd for 261.1467) in the HR-ESIMS, which required four degrees of unsaturation. The IR absorptions at 3341 and 1674 cm^−1^ indicated the presence of hydroxyl (OH) and carbonyl (CO) groups, respectively. The UV maximum at 290 nm and ^13^C NMR data indicated typical α-pyrone moiety [12,18]. The ^13^C NMR and HSQC spectra displayed three oxygenated quaternary carbons (δ_C_ 164.8–169.9), an olefinic methine carbon (δ_C_ 102.0), an sp^2^ quaternary carbon (δ_C_ 98.7), five methylene carbons (δ_C_ 28.1–40.1), an sp^3^ methine carbon (δ_C_ 29.7), a methyl carbon (δ_C_ 8.4) and an isomethyl carbon (δ_C_ 23.1) (Table 1). Analysis of the ^1^H–^1^H COSY spectrum suggested two spin systems: one from H_2_-7 at δ_H_ 2.46 to H_2_-8 at δ_H_ 1.64 and another from H_2_-9 at δ_H_ 1.34 to H_3_-13 at δ_H_ 0.88. Their connectivity with C-9 (δ_C_ 30.4) was established by a long-range HMBC correlation of H_2_-8 with C-9 (Figure 2), constructing an aliphatic chain. The position of the methyl group (δ_H_ 1.84, s) at C-3 was readily determined by its HMBC correlations with two oxygenated quaternary carbons C-2 (δ_C_ 169.9) and C-4 (δ_C_ 169.5), and as well as with the quaternary carbon C-3 (δ_C_ 98.7). Similarly, the olefinic methine proton (δ_H_ 5.96, s, H-5) showed HMBC cross-peaks with C-3, C-4, C-6 and C-7. From these HMBC correlations, together with the fact that **1** needed to form a ring to satisfy the unsaturation number, an α-pyrone ring was constructed (Figure 2). In addition, the HMBC correlation between H-5 and C-7 confirmed the connectivity of the α-pyrone ring to the aliphatic chain (Figure 2). From these data analysis, the structure of **1** was determined as a previously unreported 3,4,6-trisubstituted α-pyrone, and **1** was named violapyrone H.

**Table 1:** ^1^H and ^13^C NMR data of **1** and **2** in CD_3_OD.

Position	1		2	
	δ_C_, Type	δ_H_, Mult. (*J* in Hz)	δ_C_, Type	δ_H_, Mult. (*J* in Hz)
2	169.9, C		169.4, C	
3	98.7, C		98.9, C	
4	169.5, C		168.9, C	
5	102.0, CH	5.96, s	101.6, CH	5.97, s
6	164.8, C		164.9, C	
7	34.4, CH_2_	2.46, t (7.5)	34.4, CH_2_	2.46, t (7.5)
8	28.1, CH_2_	1.64, m	28.1, CH_2_	1.64, m
9	30.4, CH_2_	1.34	30.3, CH_2_	1.33
10	28.3, CH_2_	1.34	30.2, CH_2_	1.35
11	40.1, CH_2_	1.19, m	33.0, CH_2_	1.30, m
12	29.7, CH	1.53, m	23.8, CH_2_	1.31, m
13	23.1, CH_3_ (× 2)	0.88, d (6.5)	14.5, CH_3_	0.90, t (6.5)
3-Me	8.4, CH_3_	1.84, s	8.4, CH_3_	1.85, s

Violapyrone I (**2**) was also obtained as a yellowish amorphous solid and the molecular formula was determined to be C_13_H_20_O_3_ from the [M + Na]^+^ peak at *m/z* 247.1313 (calcd for 247.1310) in the HR-ESIMS. Preliminary, the NMR analysis showed a close similarity between the spectra of **1** and **2** (Table 1). However, the differences between these compounds were figured out from the molecular weight (CH_2_ less than **1**) and the observation of different splitting pattern of the methyl signal H-13 at δ_H_ 0.90 (t, 6.5 Hz). In addition, a lack of one methyl carbon was observed in the ^13^C NMR data of **2** compare to **1**. A detailed analysis of 1D and 2D spectra of **2** revealed the existence of a rigid α-pyrone ring same to **1**. Furthermore, an aliphatic chain was assigned by COSY and HMBC correlations, consisting of 6 methylenes with a terminal methyl proton H-13 resonated at δ_H_ 0.90 (t, 6.5 Hz). Finally, the aliphatic chain was connected to the α-pyrone ring and complete assignments of the atoms in the structure of violapyrone I (**2**) were achieved by ^1^H–^1^H COSY and HMBC experiments. Thus, the structure of **2** was determined as a new 3,4,6-trisubstituted α-pyrone, and **2** was named violapyrone I.

The structures of **3** and **4** were determined straightforward as they were very similar to those of **1** and **2**, and identified as the previously reported violapyrones B and C, respectively, by the comparison of their NMR results, MS data and optical rotation values with the literature (Supplementary Information) [18]. However, the absolute stereochemistry at C-11 of violapyrone C (**4**) was not determined in the previous report [18]. To determine the stereochemistry of C-11 in **4**, we synthesized both (*S*)- and (*R*)-violapyrones C [19]. Optical rotation values of (*S*)- and (*R*)-violapyrones C were [α]_D_^27^ +49° (*c* 0.1, MeOH) and [α]_D_^27^ −53° (*c* 0.1, MeOH), respectively. The absolute stereochemistry of C-11 in violapyrone C (**4**) was determined to be *S*, because the optical rotation value [α]_D_^27^ +50° (*c* 0.1, MeOH) and ^1^H and ^13^C NMR data were consistent with those of synthetic (*S*)-violapyrone C (Supplementary Information).

### 2.3. Cytotoxic Properties

The cytotoxicity of violapyrones H (**1**), I (**2**), B (**3**) and C (**4**) was assessed by sulforhodamine B (SRB) assay [20] using human cancer cell lines. Violapyrones (**1**–**4**) showed growth inhibitory activity against cancer cell lines at the concentrations less than 26.12 μg/mL (Table 2). Recently, violapyrones (A–G) were reported to have antibacterial activities, but did not show any cytotoxicity against five cancer cell lines (BGC-823, gastric carcinoma; Hep-G2, liver carcinoma; NCI-H460, lung carcinoma; HeLa, cervical carcinoma; HCT-116, colon carcinoma) when tested using MTT method [18]. However, we found the cytotoxicity of violapyrones H (**1**) and I (**2**) as well as B (**3**) and C (**4**) against human cancer cell lines (HeLa; ACHN, renal carcinoma; HCT-15 and HCT-116, colon carcinomas; MDA-MB-231, breast carcinoma; NCI-H23 and NCI-H460, lung carcinomas; NUGC-3, stomach carcinoma; Hep-G2; PC-3, prostate carcinoma). Especially, compound **1** showed the highest activity against HCT-15 cell line with a GI_50_ value of 1.10 μg/mL. Moreover, it may be noteworthy that each compound has structural similarity, but showed different activities. Our results suggested that the length of the aliphatic side chain and the position of the methyl group affected the activity. Furthermore, violapyrones having an isomethyl group in the alkyl side chain showed better activity than others. Violapyrones (A–G) also showed quite similar tendency in their antibacterial activities [18].

**Table 2:** Growth Inhibition (GI_50_, μg/mL) of **1**–**4** against a Panel of Human Tumor Cell Lines.

Cell Lines	GI_50_^a^ (μg/mL)				
	1	2	3	4	ADR ^b^
Cervical cancer: HeLa	25.05	5.54	18.12	9.91	0.09
Renal cancer: ACHN	1.79	5.42	1.18	1.55	0.04
Colon cancer: HCT-15	1.10	3.38	2.01	5.22	0.08
Colon cancer: HCT-116	8.99	18.08	15.83	26.12	0.09
Breast cancer: MDA-MB-231	1.51	6.29	1.80	4.94	0.99
Lung cancer: NCI-H23	1.24	3.47	1.90	3.24	0.04
Lung cancer: NCI-H460	4.45	21.04	6.37	10.80	0.07
Stomach cancer: NUGC-3	1.27	3.36	2.24	4.02	0.12
Liver cancer: Hep-G2	2.30	14.60	2.04	3.96	0.08
Prostate cancer: PC-3	1.37	5.44	1.40	2.06	0.06

^a^ GI_50_ values are the concentration corresponding to 50% growth inhibition; ^b^ ADR: adriamycin as standard.

## 3. Experimental Section

### 3.1. General Experimental Procedures

Optical rotation was measured on a JASCO DIP-1000 digital polarimeter (JASCO Corporation, Tokyo, Japan), with a 1 cm cell. UV spectra were obtained on a Shimadzu UV-1650PC spectrophotometer (Shimadzu Corporation, Kyoto, Japan). IR spectra were recorded on a JASCO FT/IR-4100 spectrophotometer, (JASCO Corporation, Tokyo, Japan). Nuclear magnetic resonance (NMR) spectra, including ^1^H–^1^H COSY, HSQC and HMBC experiments, were collected on a Varian Unity 500 spectrometer (Varian Inc., Palo Alto, CA, USA) operating at 500 MHz (^1^H) and 125 MHz (^13^C) with chemical shifts given in ppm (δ). High-resolution ESI mass spectroscopy was recorded on a hybrid ion-trap time-of-flight mass spectrometer (SYNAPT G2, Waters Corporation, Milford, CT, USA). High performance liquid chromatography (HPLC) was conducted with a PrimeLine pump (Analytical Scientific Instruments, Inc., El Sobrante, CA, USA) with RI-71 refractive index detector (Shodex, Shoko Scientific Co. Ltd., Yokohama, Japan). Open column chromatography was carried out over a Pyrex glass (300 mm × 50 mm). RP-C_18_ silica gel (YMC-Gel ODS-A, 12 nm S-75 μm) was used for column chromatography. All solvents used were either spectral grade of distilled prior to use. Continuous centrifugation was done on a centrifugal separator (Kansai Centrifugal Separator Manufacturing Co. Ltd., Osaka, Japan).

### 3.2. Isolation and Identification of the Strain 112CH148

The strain designated as 112CH148 was isolated from a crown-of-thorns starfish, *Acanthaster planci*, collected from Chuuk, Federated States of Micronesia in 2011. A portion of sample was rinsed with sterilized sea water under aseptic condition and then put on Bennett’s agar plates (1% dextrose, 0.2% tryptone, 0.1% yeast extract, 0.1% beef extract, 0.5% glycerol, 1.7% agar, salinity 32 g/L, pH 7.02 before sterilization). The plates were incubated for 12 days at 28 °C, and the resulting colony of the strain 112CH148 was isolated and maintained on Bennett’s agar plates. The strain was identified as *Streptomyces* sp. on the basis of 16S rRNA sequence analysis. The sequence was deposited in the GenBank under the accession number KJ419328. This strain is currently preserved in the Microbial Culture Collection, KIOST, with the name of *Streptomyces* sp. 112CH148 under the curatorship of Hee Jae Shin.

### 3.3. Seed and Mass Cultures of the Strain

The seed and mass culture were carried out in Bennett’s medium (1% dextrose, 0.2% tryptone, 0.1% yeast extract, 0.1% beef extract, 0.5% glycerol, salinity 32 g/L, pH 7.02 before sterilization). The 200 mL medium was dispensed in a 500 mL conical flask and sterilized. A single colony of the strain from the agar plate was inoculated aseptically into the flask and incubated at 28 °C for 2 days on a rotary shaker at 120 rpm. An aliquot (0.2% v/v) from the seed culture was inoculated aseptically into 2 L flasks (total 24 flasks) containing 1.3 L medium and a 20 L fermenter containing 18 L of sterilized culture medium, respectively. The production culture was incubated under the same conditions as the seed culture for 7 days and then harvested. The mass cultures were carried out three times.

### 3.4. Extraction and Isolation of Compounds

The culture broth (total 150 L) was harvested by high speed centrifugation (60,000 rpm) and then extracted with EtOAc (2 times). The EtOAc extract was evaporated to obtain crude extract (13.85 g). The crude extract was subjected to ODS open column chromatography followed by stepwise gradient elution with MeOH/H_2_O (v/v) (1:4, 2:3, 3:2, 4:1 and 100:0) as eluent. The subfraction eluted with MeOH/H_2_O (4:1) was again applied to an ODS open column chromatography with a MeOH/H_2_O solvent system (6:4, 7:3, 8:2 and 100:0). The fraction eluted with MeOH/H_2_O (8:2) was purified by a reversed-phase HPLC (YMC ODS-A column, 250 × 10 mm i.d, 5 μm; 70% MeOH in H_2_O; flow rate: 2.0 mL/min; detector: RI) to yield pure compounds **1** (1.9 mg, *t*_R_ 32.5 min) and **4** (9.0 mg, *t*_R_ 31.0 min). The subfraction eluted with MeOH/H_2_O (7:3) was also purified by a RP-HPLC (YMC ODS-A column, 250 × 10 mm i.d, 5 μm; 60% MeOH; flow rate: 2.0 mL/min; detector: RI) to get compounds **2** (2.7 mg, *t*_R_ 30.5 min) and **3** (5.0 mg, *t*_R_ 34.0 min).

Violapyrone H (**1**): Yellowish amorphous solid; UV (MeOH) λ_max_ (log ε) 290 (0.52) nm; IR (MeOH) ν_max_ 3341 (br), 2935, 1674 cm^−1^; ^1^H and ^13^C NMR data (CD_3_OD), Table 1; HR-ESIMS *m/*z 261.1466 [M + Na]^+^.

Violapyrone I (**2**): Yellowish amorphous solid; UV (MeOH) λ_max_ (log ε) 289 (0.70) nm; IR (MeOH) ν_max_ 3345 (br), 2926, 1670 cm^−1^; ^1^H and ^13^C NMR data (CD_3_OD), Table 1; HR-ESIMS *m/*z 247.1313 [M + Na]^+^.

Violapyrone B (**3**): Yellowish amorphous solid; UV (MeOH) λ_max_ (log ε) 286.5 (1.34) nm; IR (MeOH) ν_max_ 3347 (br), 2943, 1674 cm^−1^; ^1^H NMR (CD_3_OD) δ_H_ 5.97 (1H, s, H-5), 2.45 (2H, t, *J* = 7.5 Hz, H-7), 1.85 (3H, s, Me-3), 1.62 (2H, m, H-8), 1.54 (1H, m, H-11), 1.35 (2H, m, H-9), 1.23 (2H, m, H-10), 0.88 (6H, d, *J* = 7.0 Hz, H-12); ^13^C NMR (CD_3_OD) δ_C_ 169.4 (C-2), 168.8 (C-4), 164.9 (C-6), 101.5 (C-5), 98.9 (C-3), 39.9 (C-10), 34.4 (C-7), 29.2 (C-11), 28.3 (C-8), 27.9 (C-9), 23.1 (C-12), 8.4 (Me-3); HR-ESIMS *m/*z 225.1485 [M + H]^+^.

Violapyrone C (**4**): Yellowish amorphous solid; [*α*]_D_^27^ +50 (*c* 0.1, MeOH); UV (MeOH) λ_max_ (log ε) 288.0 (0.91) nm; IR (MeOH) ν_max_ 3343 (br), 2925, 1674 cm^−1^; ^1^H NMR (CD_3_OD) δ_H_; 5.98 (1H, s, H-5), 2.47 (2H, t, *J* = 7.5 Hz, H-7), 1.85 (3H, s, Me-3), 1.62 (2H, m, H-8), 1.36 (2H, H-9), 1.34 (1H, H_a_-12), 1.13 (1H, H_b_-12), 1.33 (1H, H_a_-10), 1.17 (1H, H_b_-10), 1.32 (1H, H-11), 0.88 (3H, t, *J* = 6.5 Hz, H-13), 0.87 (3H, d, *J* = 6.0 Hz, Me-11); ^13^C NMR (CD_3_OD) δ_C_; 169.5 (C-4), 169.2 (C-2), 164.9 (C-6), 101.8 (C-5), 98.8 (C-3), 37.5 (C-12), 35.7 (C-11), 34.4 (C-7), 30.7 (C-10), 28.4 (C-8), 27.6 (C-9), 19.7 (Me-11), 11.9 (C-13), 8.4 (Me-3); HR-ESIMS *m/z* 261.1461 [M + Na]^+^.

### 3.5. Cytotoxicity Test by SRB Assay

Human cancer cell lines, HeLa (cervix), ACHN (renal), HCT-15 (colon), HCT-116 (colon), MDA-MB-231 (breast), NCI-H23 (lung), NCI-H460 (lung), NUGC-3 (stomach), Hep-G2 (liver) and PC-3 (prostate), were purchased from American Type Culture Collection (Manassas, VA). The cell lines were cultured RPMI 1640 supplemented with 10% fetal bovine serum (FBS). Cell cultures were maintained at 37 °C under a humidified atmosphere of 5% CO_2_. The growth inhibition assay against human cancer cell lines was carried out according to a sulforhodamine B (SRB) assay [20]. In brief, 8000 cells/well were seeded in a 96-well plate. Next day, the cells were treated with violapyrones H (**1**), I (**2**), B (**3**) and C (**4**) including vehicle control (0.1% DMSO) and positive control (adriamycin). After being incubated for 48 hours, cultures were fixed with 50% trichloroactetic acid (50 μg/mL) and stained with 0.4% sulforhodamine B in 1% acetic acid. Unbound dye was removed by washing with 1% acetic acid, and protein-bound dye was extracted with 10 mM Tris base (pH 10.5) for determination of optical density. The absorbance at 540 nm was determined using a VersaMax microplate reader (Molecular Devices, LLC, Sunnyvale, CA, USA). GI_50_ values were calculated using GraphPad Prism 4.0 software (GraphPad Software, Inc., San Diego, CA, USA).

## 4. Conclusions

As a result, we isolated two new 3,4,6-trisubstituted α-pyrone derivatives, violapyrones H (**1**) and I (**2**) and two known B (**3**) and C (**4**), from the culture broth of *Streptomyces* sp. 112CH148. These violapyrones exhibited the cytotoxicity against 10 human cancer cell lines (HeLa, ACHN, HCT-15, HCT-116, MDA-MB-231, NCI-H23, NCI-H460, NUGC-3, Hep-G2 and PC-3). Consequentially, these compounds could be new frontiers for the development of anticancer agents. Further studies are needed to clearly elucidate the mechanism of structure-activity relationship.

## Supplementary Files

Supplementary File 1 Supplementary Information (PDF, 863 KB)
