# Supplementary material for: Violapyrones H and I, New Cytotoxic Compounds Isolated from Streptomyces sp. Associated with the Marine Starfish Acanthaster planci
Source: Mar Drugs. 2014 May 30;12(6):3283–91. doi: 10.3390/md12063283 (PMC4071576; doi:10.3390/md12063283)
Supplement: Supplementary File 1 — Supplementary Information (PDF, 863 KB) [file marinedrugs-12-03283-s001.pdf]

Supplementary Information

Figure S1. ^{1}H NMR spectrum of violapyrone H (**1**) in CD_3OD (500 MHz).
Figure S2. ^{13}C NMR spectrum of violapyrone H (**1**) in CD_3OD (125 MHz).
Figure S3. HSQC spectrum of violapyrone H (**1**) in CD_3OD.
Figure S4. COSY spectrum of violapyrone H (**1**) in CD3OD.
Figure S5. HMBC spectrum of violapyrone H (**1**) in CD_3OD.
Figure S6. HR-ESIMS data of violapyrone H (**1**).
Figure S7. ^{1}H NMR spectrum of violapyrone I (**2**) in CD_3OD (500 MHz).
Figure S8. ^{13}C NMR spectrum of violapyrone I (**2**) in CD_3OD (125 MHz).
Figure S9. HSQC spectrum of violapyrone I (**2**) in CD_3OD.
Figure S10. COSY spectrum of violapyrone I (**2**) in CD_3OD.
Figure S11. HMBC spectrum of violapyrone I (**2**) in CD_3OD.
Figure S12. HR-ESIMS data of violapyrone I (**2**).
Figure S13. ^{1}H NMR spectrum of violapyrone B (**3**) in CD_3OD (500 MHz).
Figure S14. ^{13}C NMR spectrum of violapyrone B (**3**) in CD_3OD (125 MHz).
Figure S15. ^{1}H NMR spectrum of natural *(S)*-violapyrone C (**4**) in CD_3OD (500 MHz).
Figure S16. ^{1}H NMR spectrum of synthetic *(S)*-violapyrone C in CD_3OD (500 MHz).
Figure S17. ^{13}C NMR spectrum of natural *(S)*-violapyrone C (**4**) in CD_3OD (125 MHz).
Figure S18. ^{13}C NMR spectrum of synthetic *(S)*-violapyrone C in CD_3OD (125 MHz).

Figure S1. ^{1}H NMR spectrum of violapyrone H (**1**) in CD_3OD (500 MHz).

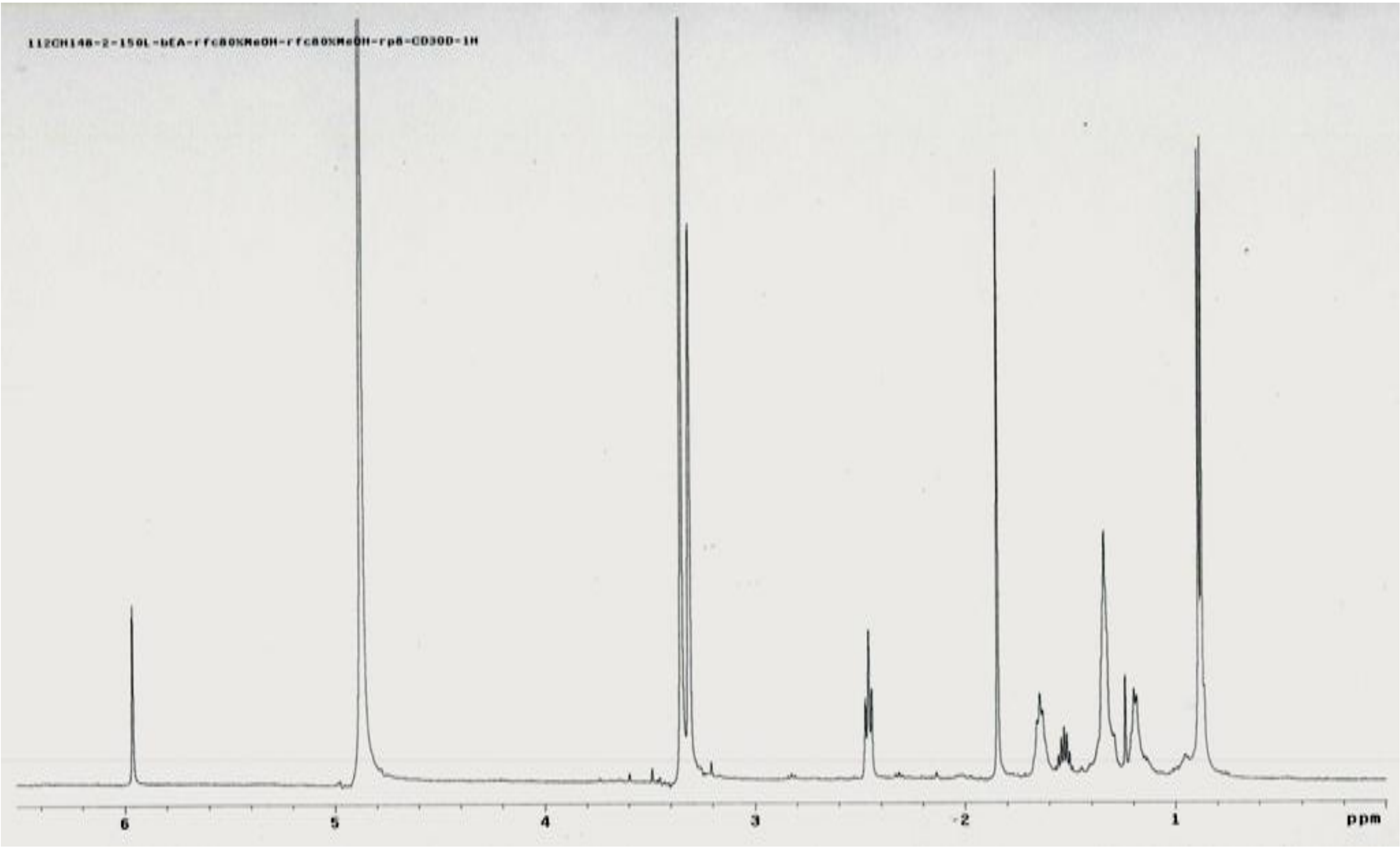

Figure S2. ^{13}C NMR spectrum of violapyrone H (**1**) in CD_3OD (125 MHz).

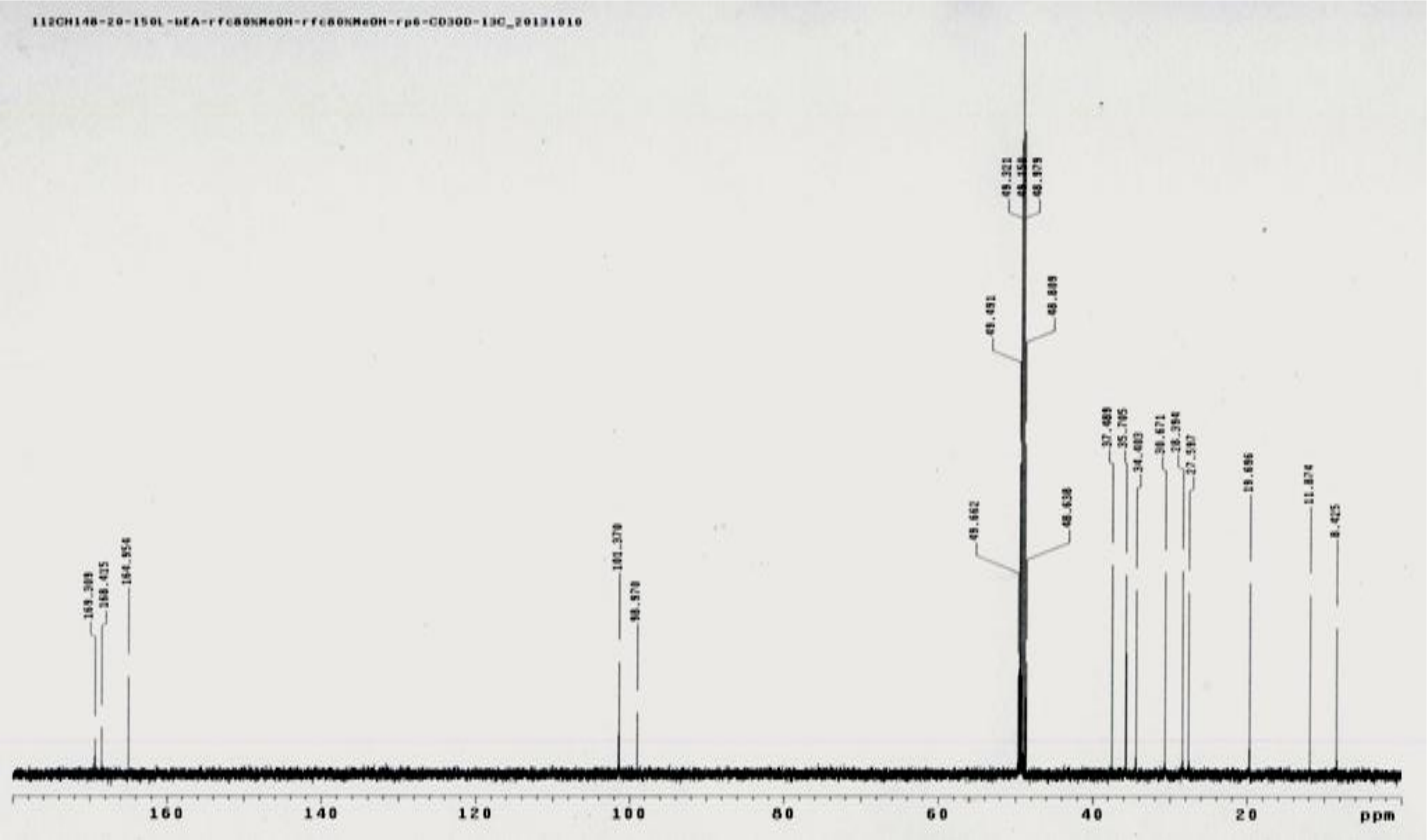

Figure S3. HSQC spectrum of violapyrone H (**1**) in CD_3OD.

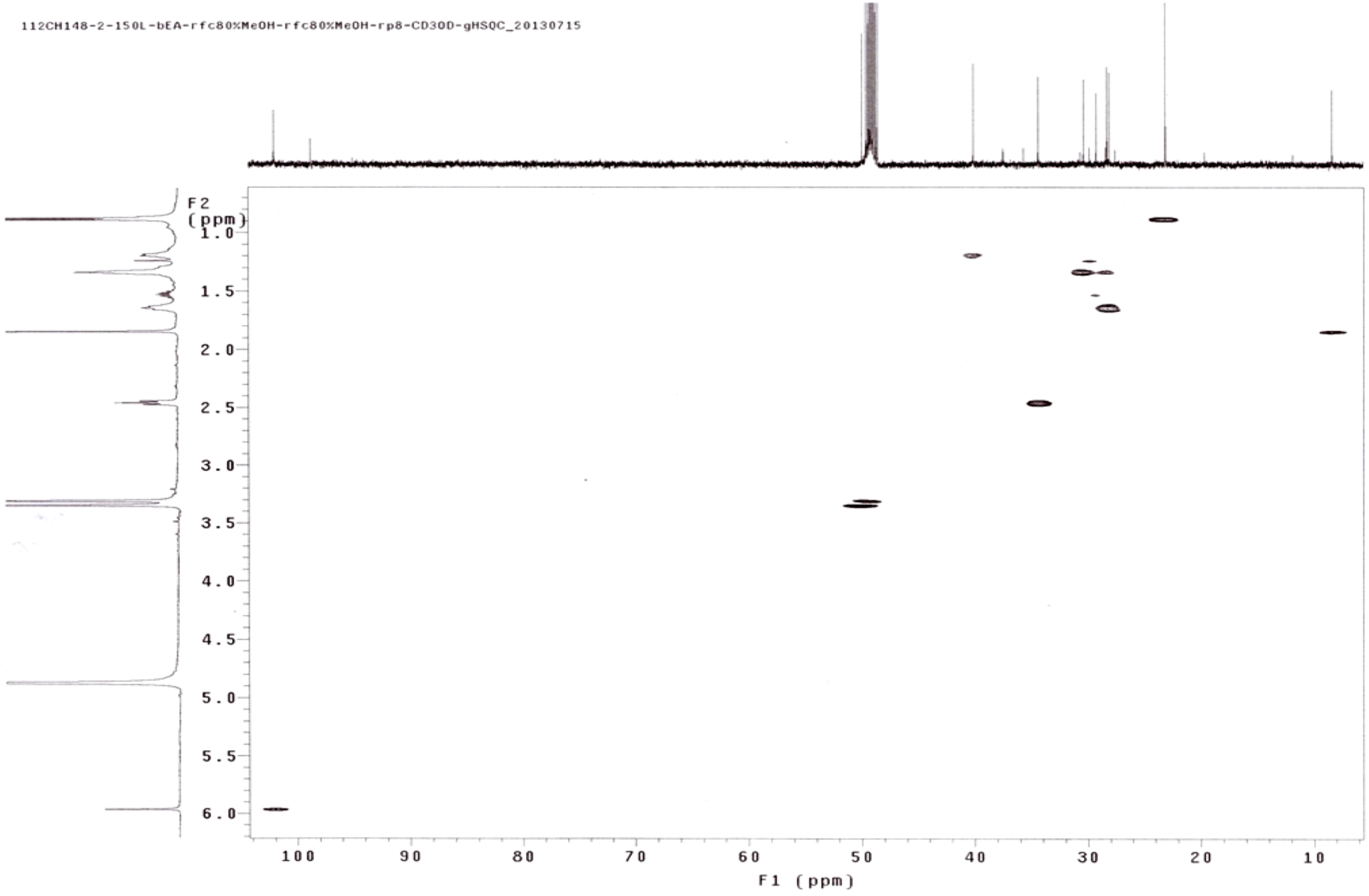

Figure S4. COSY spectrum of violapyrone H (**1**) in CD3OD.

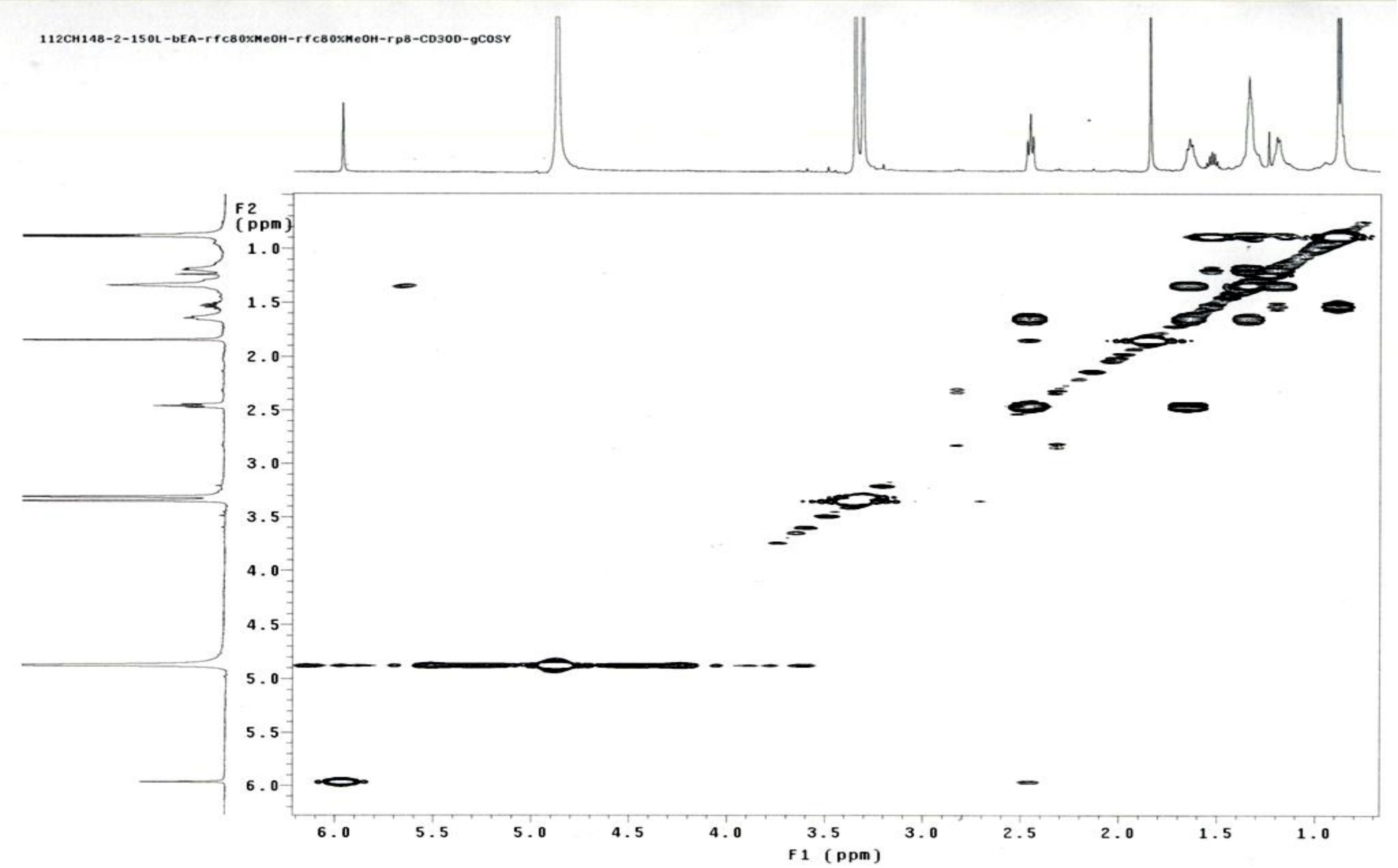

Figure S5. HMBC spectrum of violapyrone H (**1**) in CD_3OD.

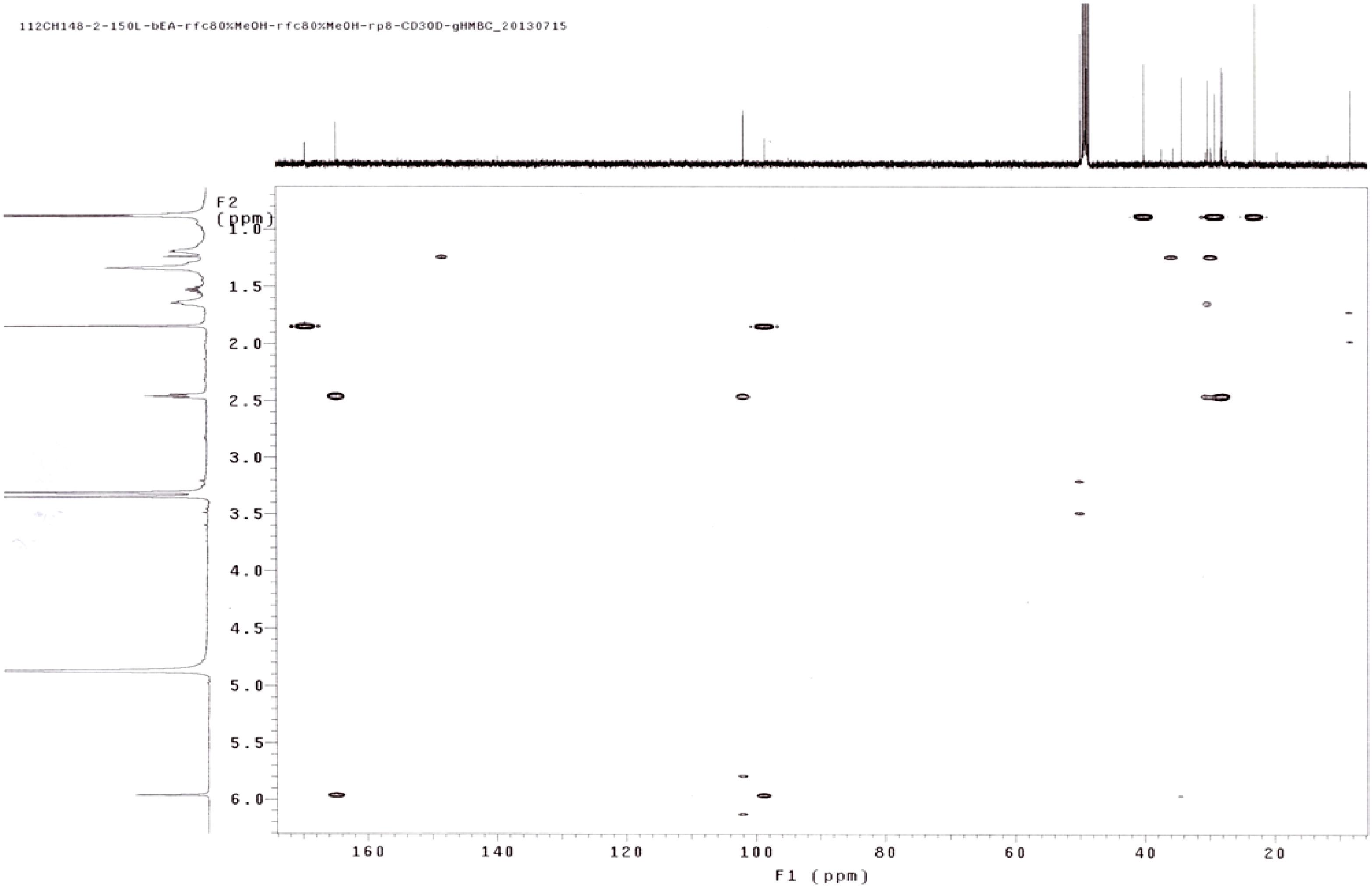

Figure S6. HR-ESIMS data of violapyrone H (**1**).

Elemental Composition Report

Single Mass Analysis
Tolerance = 10.0 PPM / DBE: min = -1.5, max = 50.0
Element prediction: Off
Number of isotope peaks used for i-FIT = 3

Monoisotopic Mass, Even Electron Ions
31 formula(e) evaluated with 1 results within limits (all results (up to 1000) for each mass)
Elements Used:
C: 1-500 H: 1-1000 O: 1-100 Na: 1-1

Minimum:				-1.5				
Maximum:		5.0	10.0	50.0				
Mass	Calc. Mass	mDa	PPM	DBE	i-FIT	Norm	Conf(%)	Formula
261.1466	261.1467	-0.1	-0.4	3.5	900.0	n/a	n/a	C14 H22 O3 Na

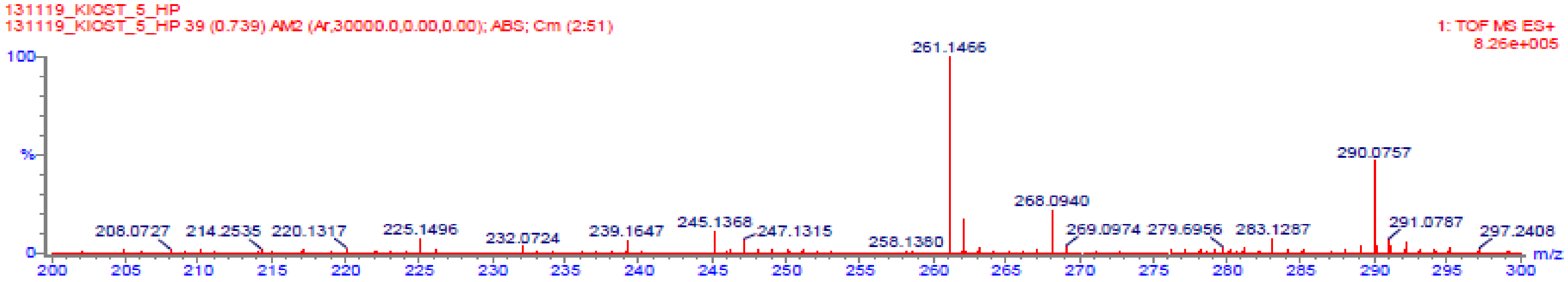

Figure S7. ^{1}H NMR spectrum of violapyrone I (**2**) in CD_3OD (500 MHz).

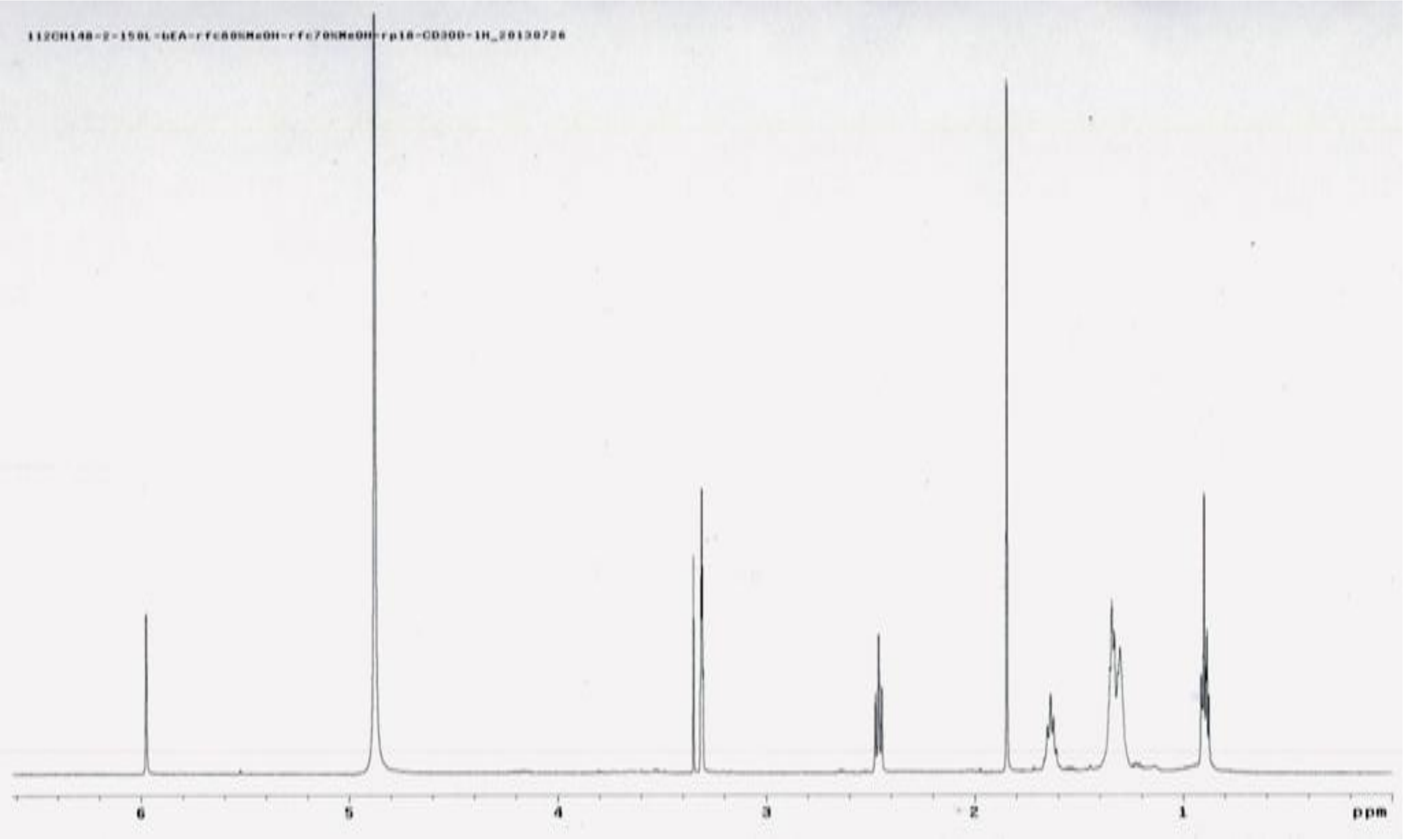

Figure S8. ^{13}C NMR spectrum of violapyrone I (**2**) in CD_3OD (125 MHz).

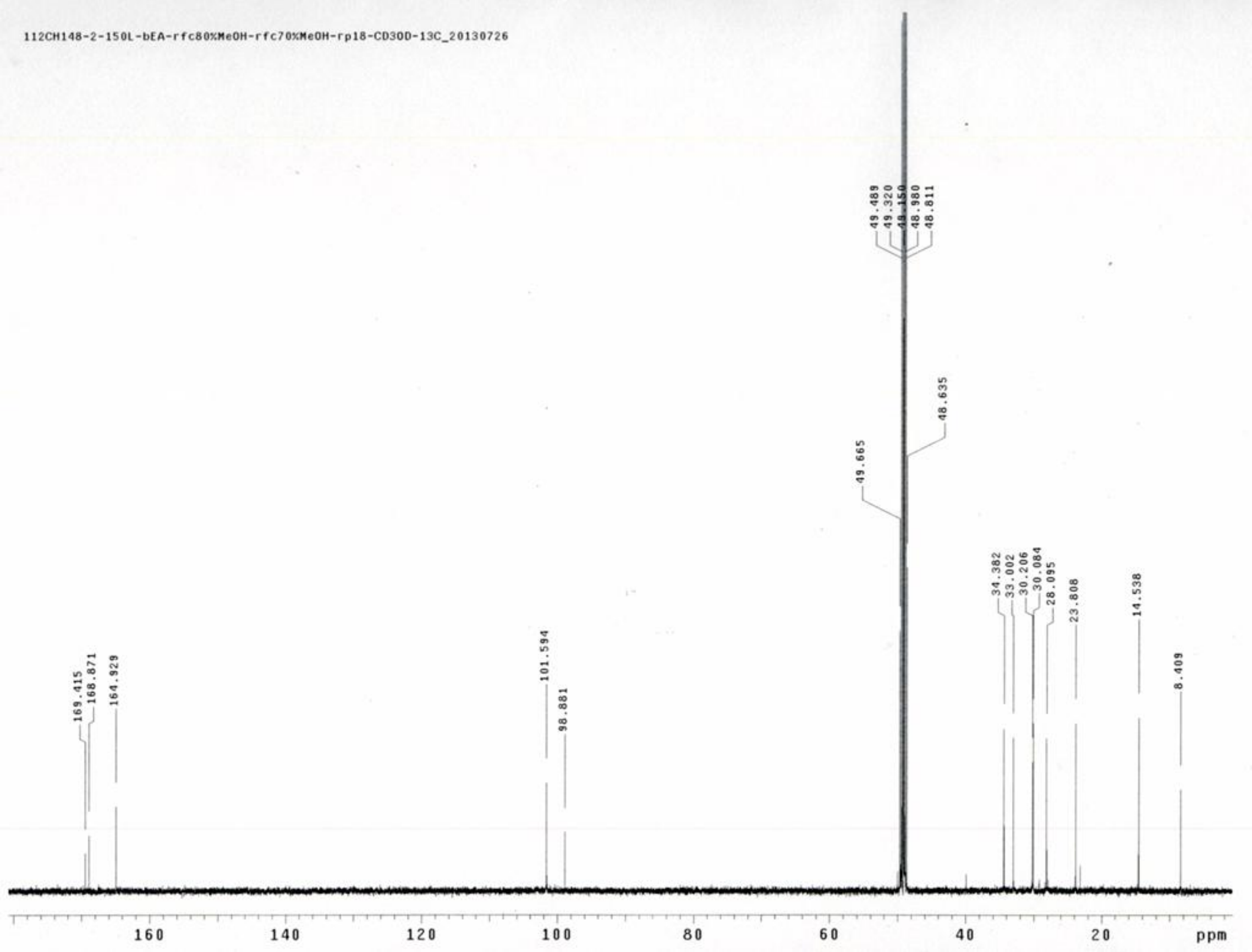

Figure S9. HSQC spectrum of violapyrone I (**2**) in CD_3OD.

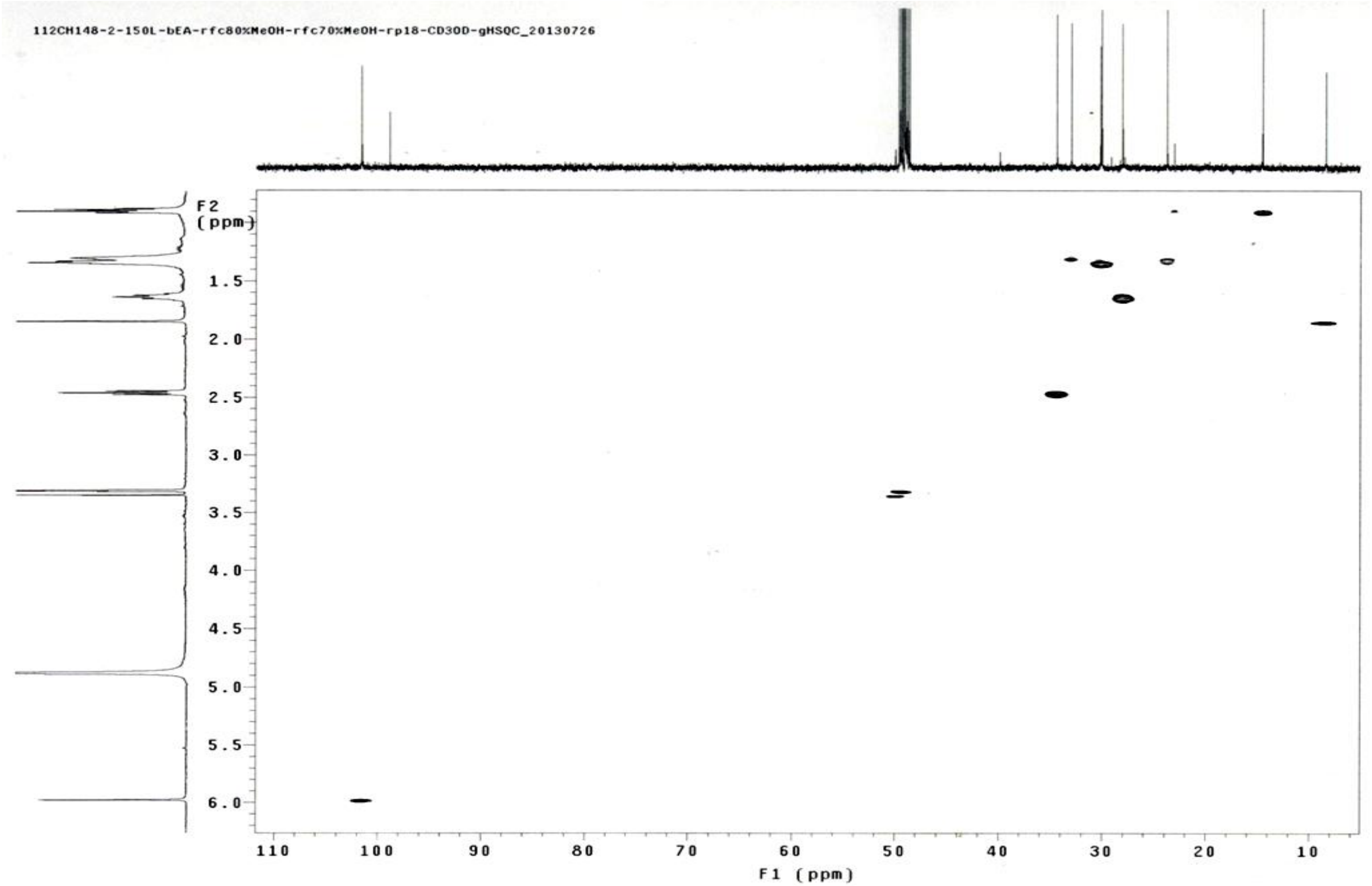

Figure S10. COSY spectrum of violapyrone I (**2**) in CD_3OD.

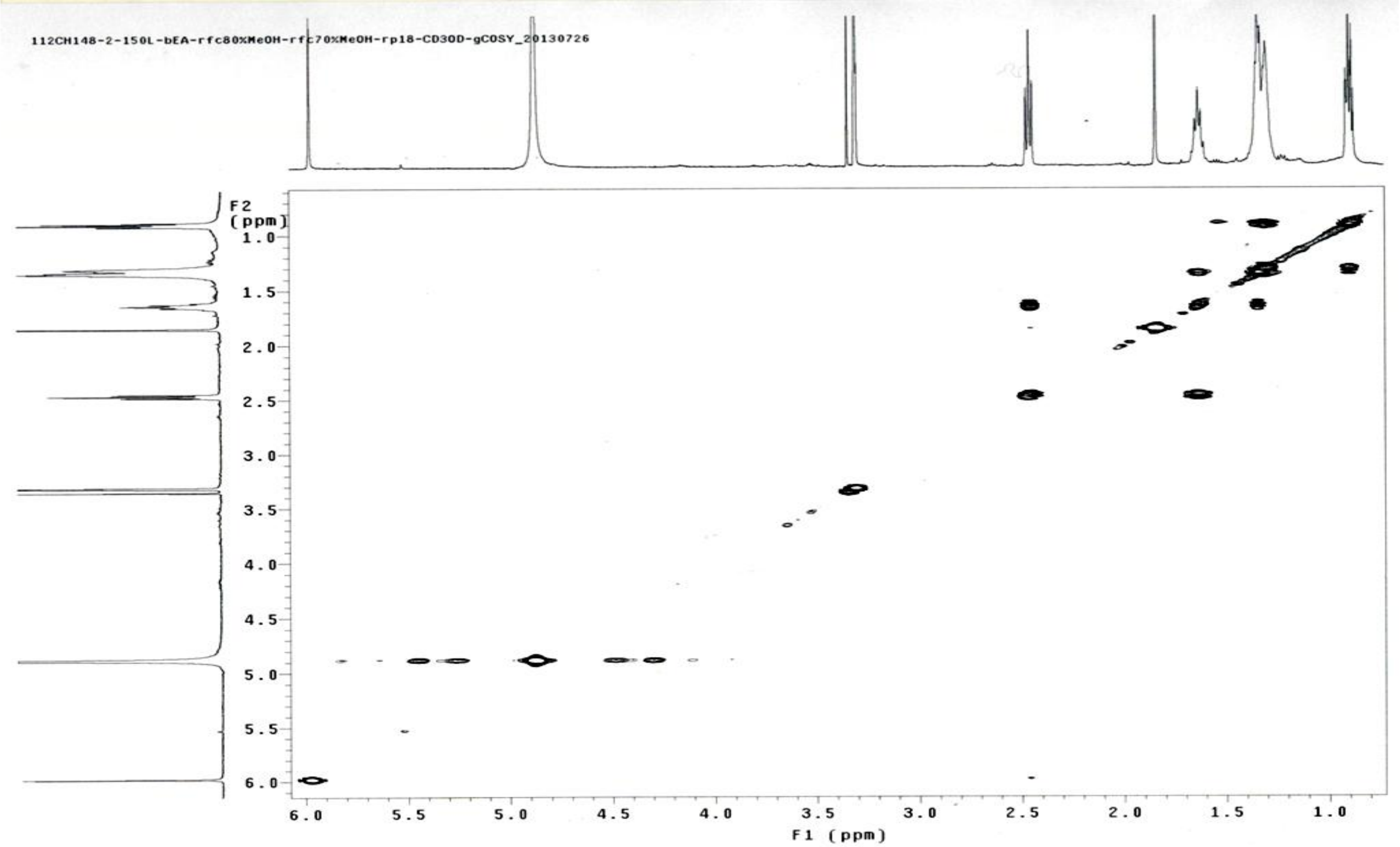

Figure S11. HMBC spectrum of violapyrone I (**2**) in CD_3OD.

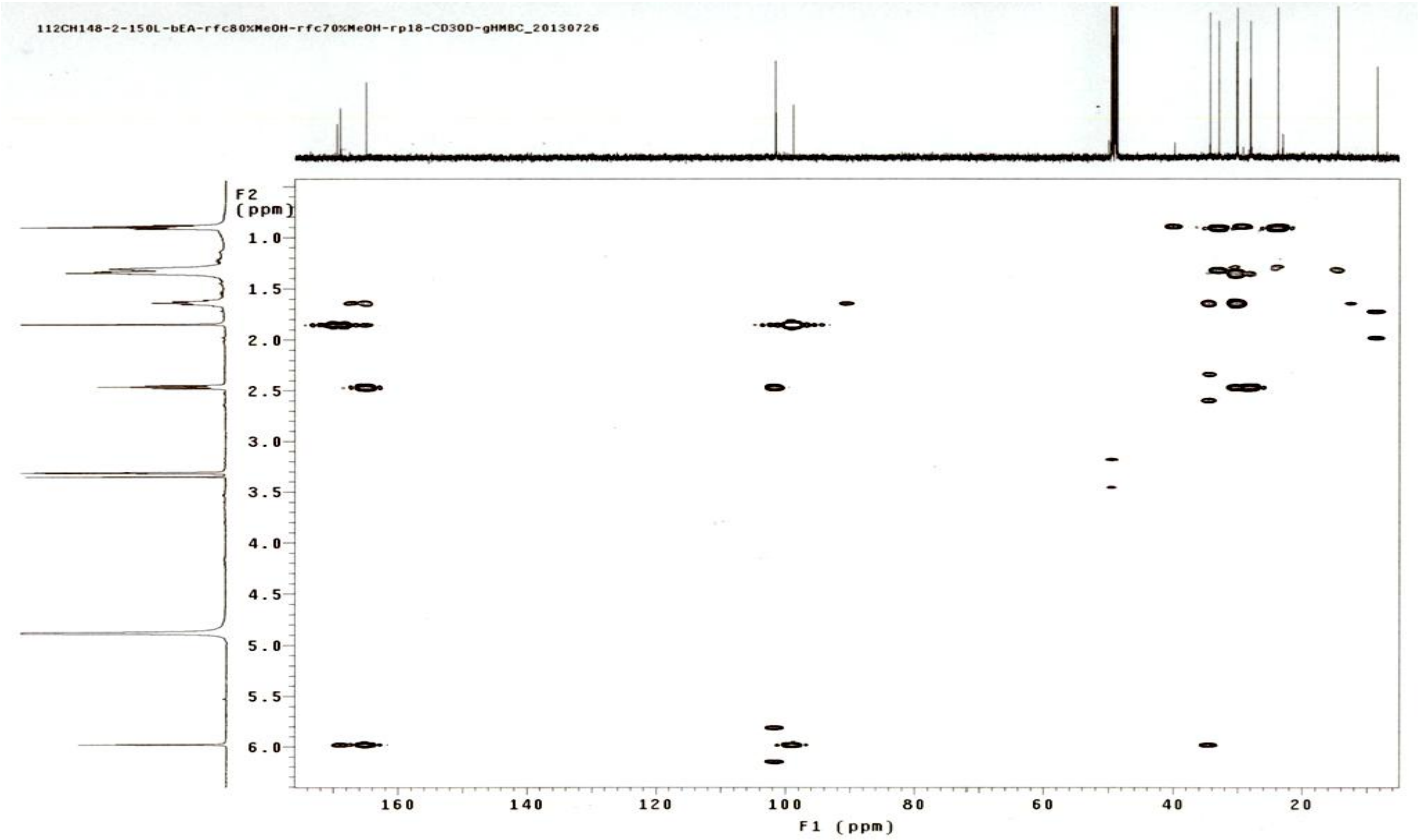

Figure S12. HR-ESIMS data of violapyrone I (**2**).

Elemental Composition Report

Single Mass Analysis
Tolerance = 50.0 PPM / DBE: min = -1.5, max = 50.0
Element prediction: Off
Number of isotope peaks used for i-FIT = 3

Mass	Calc. Mass	mDa	PPM	DBE	i-FIT	Norm	Conf(%)	Formula
247.1313	247.1310	0.3	1.2	3.5	1028.8	n/a	n/a	C13 H20 O3 Na

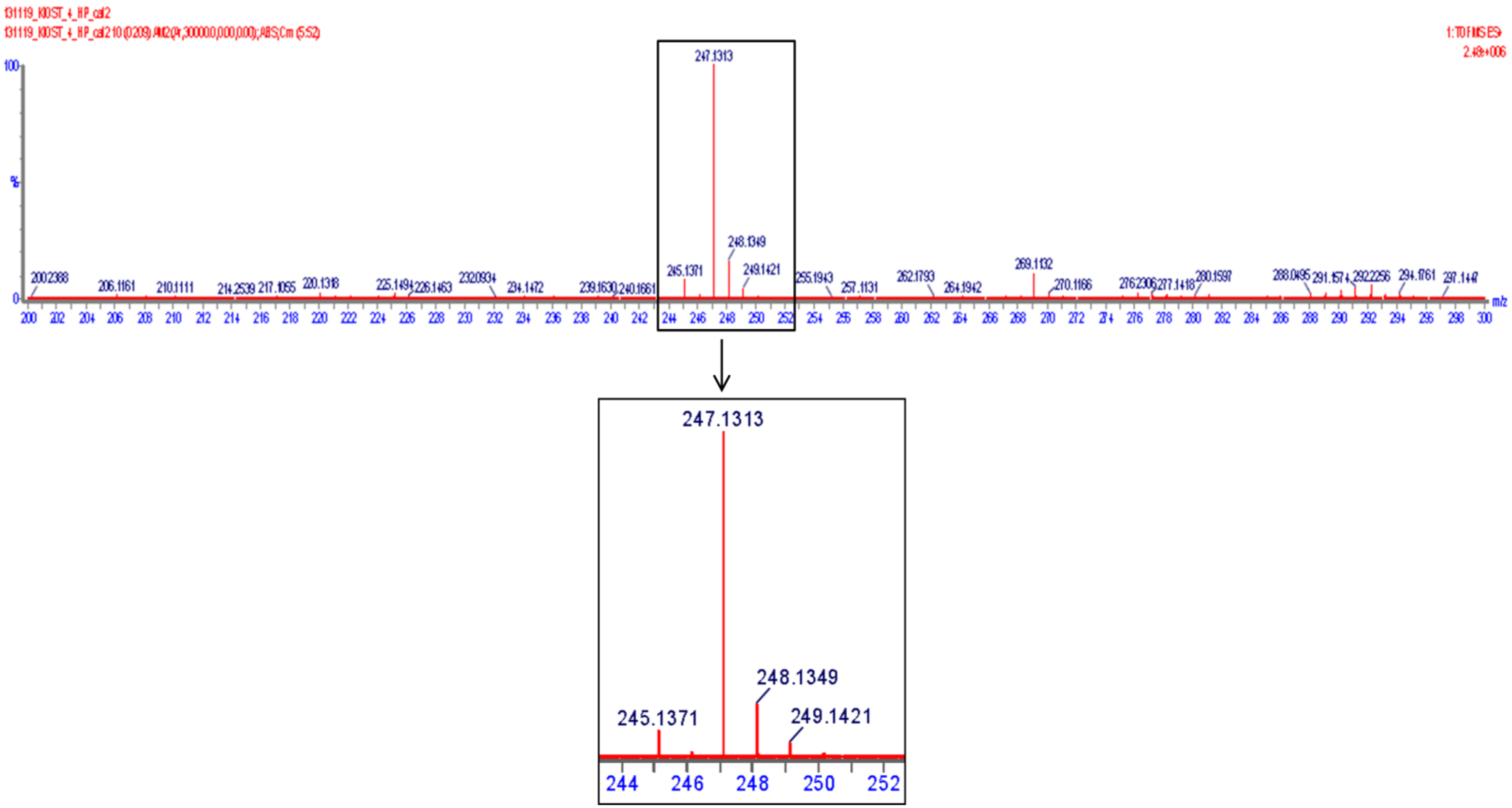

Figure S13. ^{1}H NMR spectrum of violapyrone B (**3**) in CD_3OD (500 MHz).

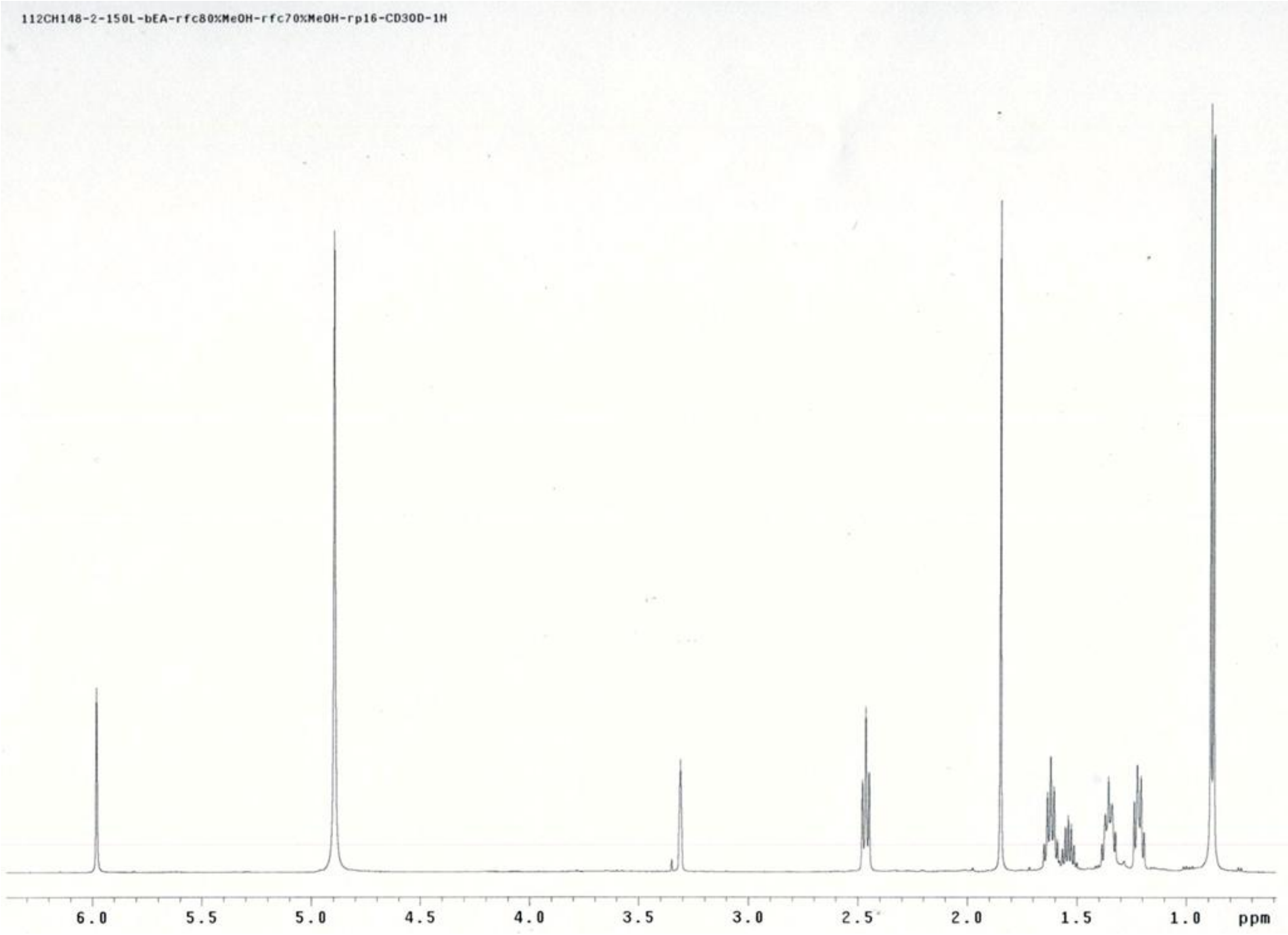

Figure S14. ^{13}C NMR spectrum of violapyrone B (**3**) in CD_3OD (125 MHz).

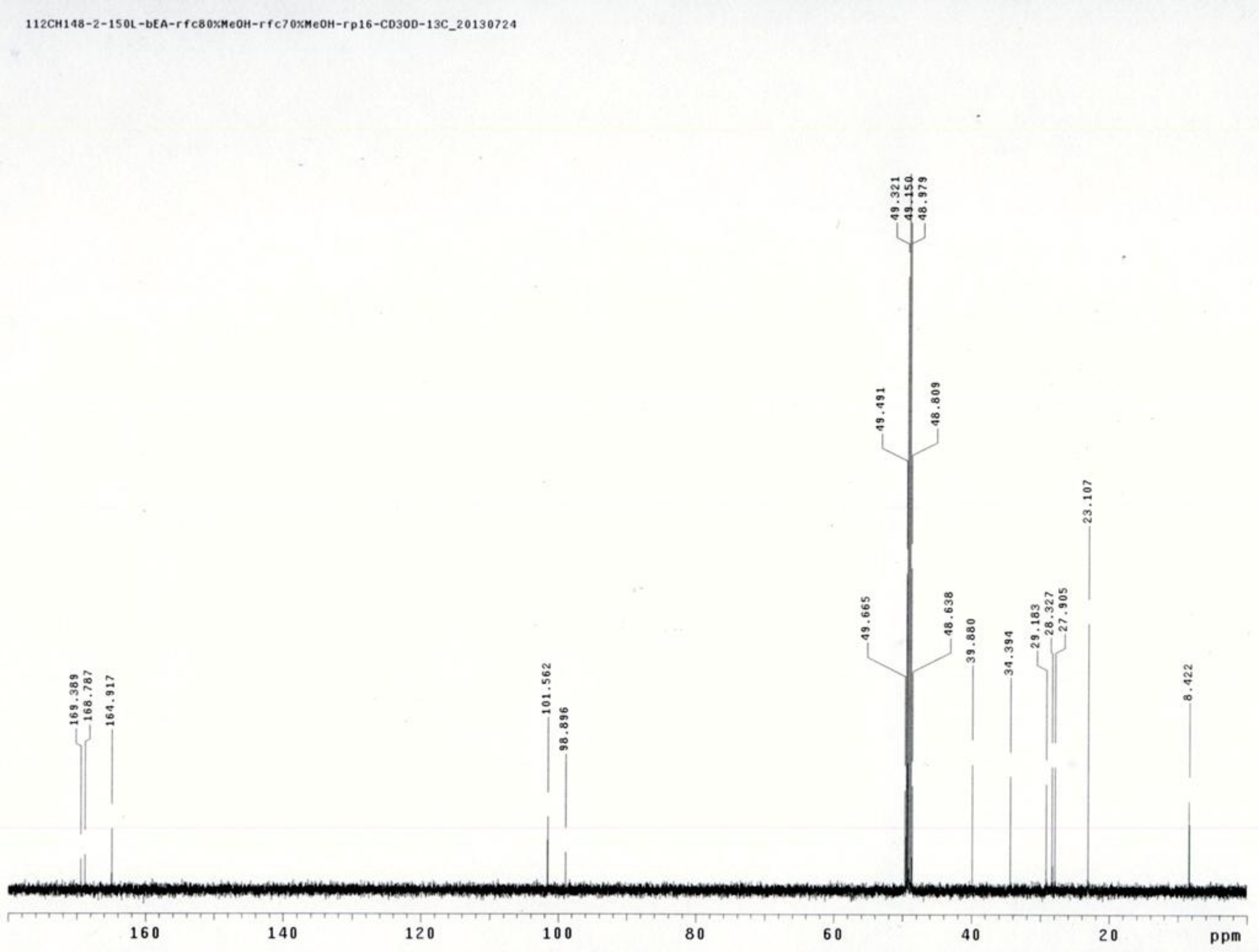

Figure S15. ^{1}H NMR spectrum of natural (*S*)-violapyrone C (**4**) in CD_3OD (500 MHz).

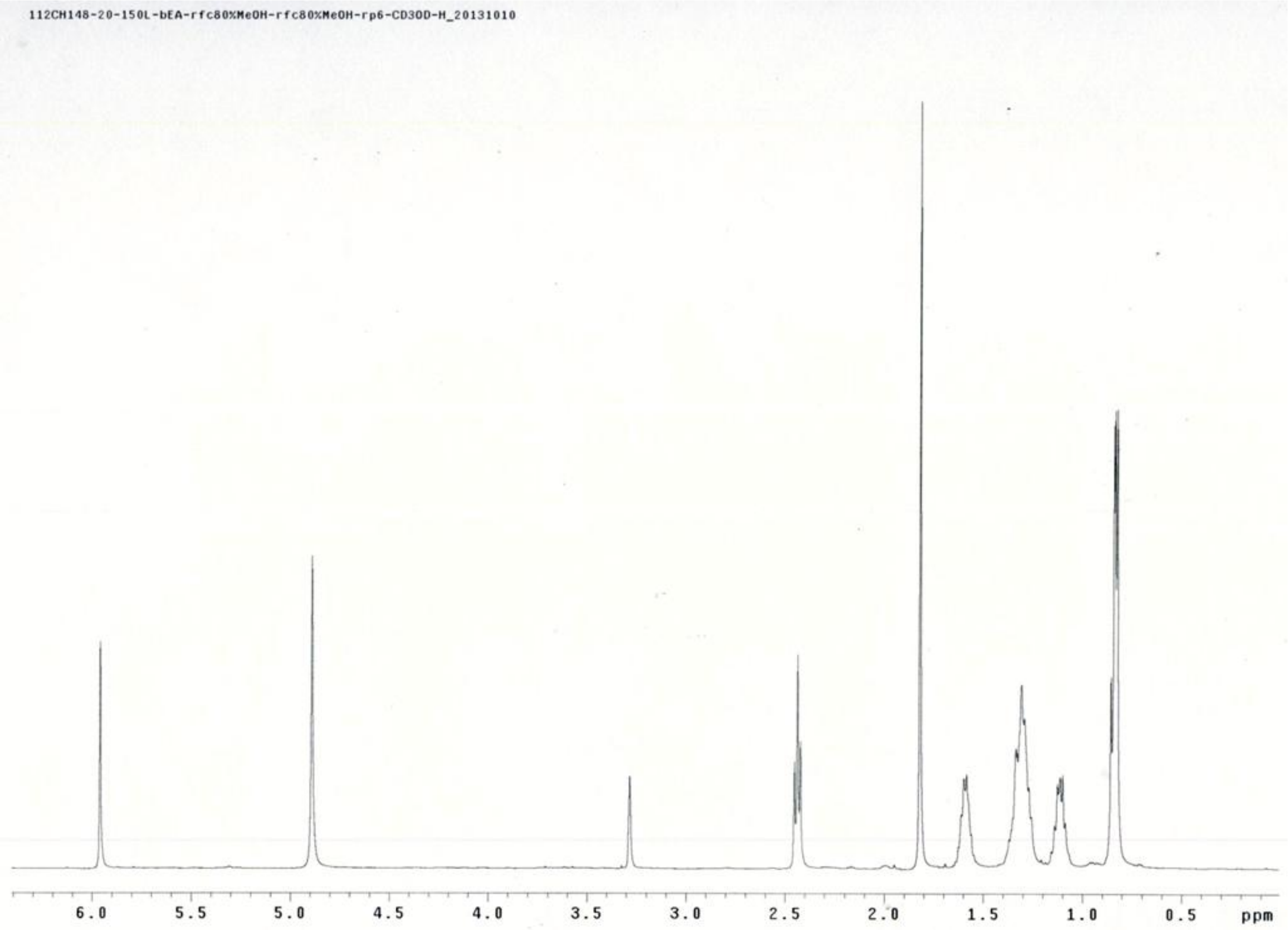

Figure S16. ^{1}H NMR spectrum of synthetic (*S*)-violapyrone C in CD_3OD (500 MHz).

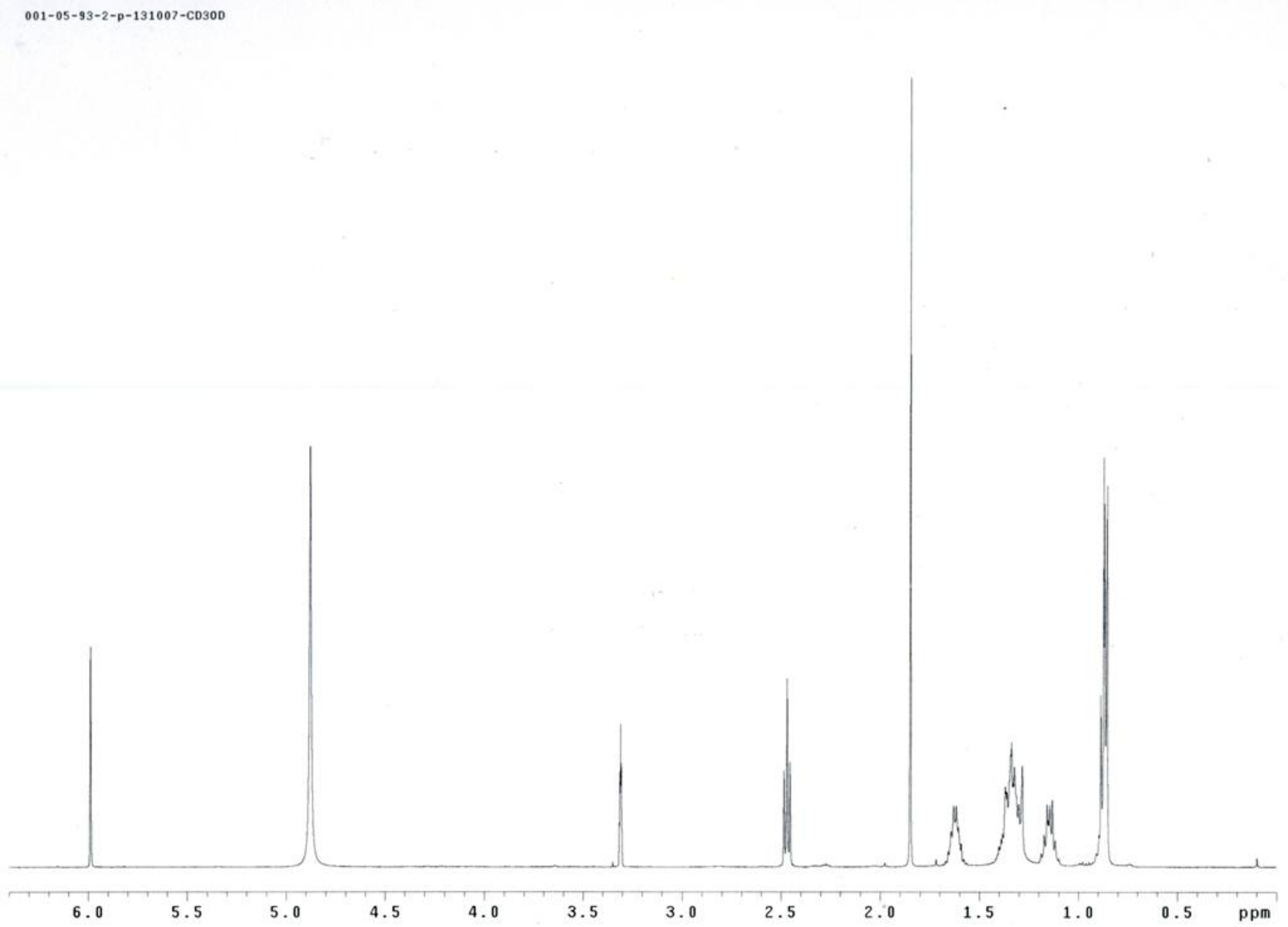

Figure S17. ^{13}C NMR spectrum of natural (*S*)-violapyrone C (**4**) in CD_3OD (125 MHz).

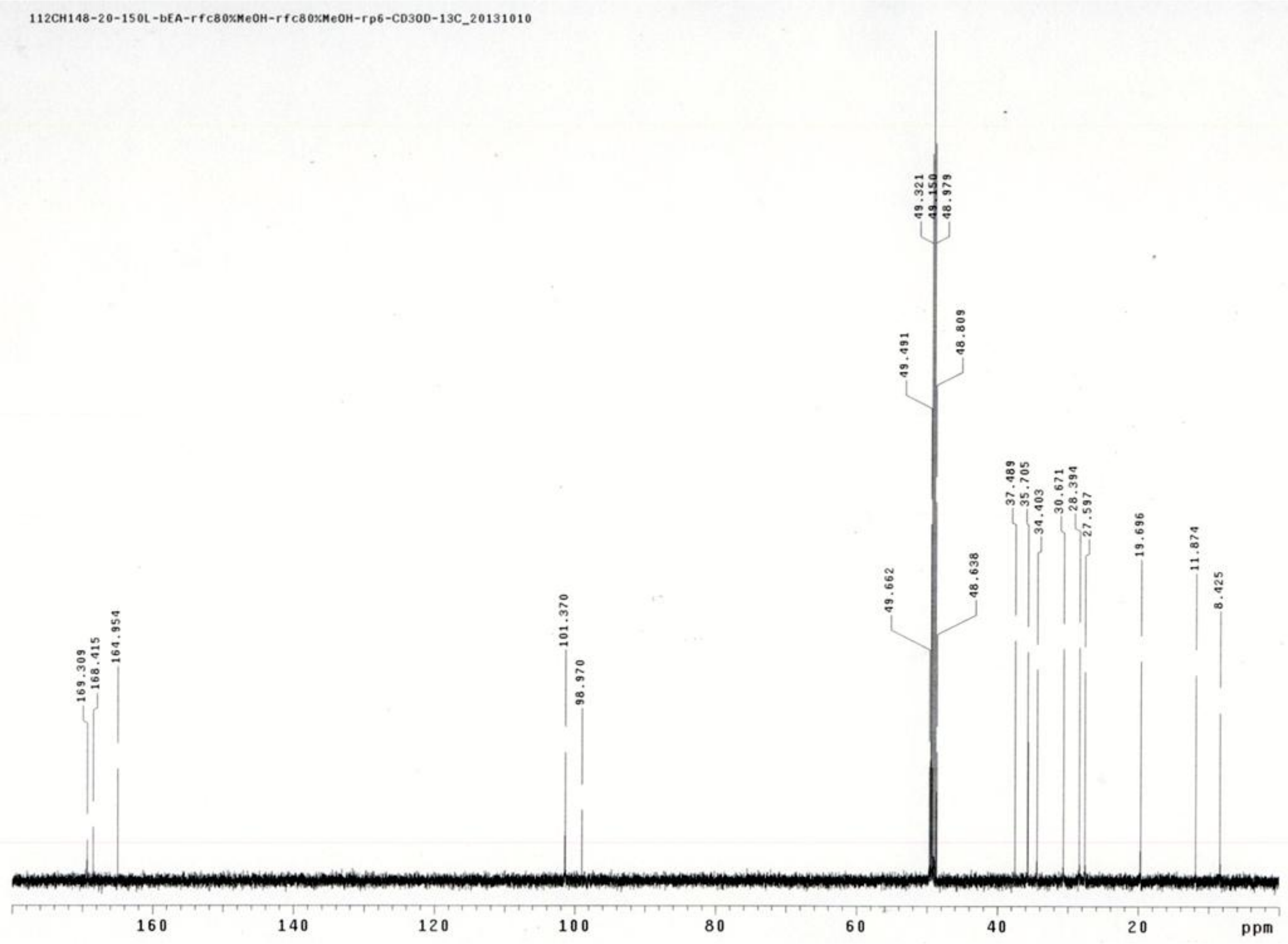

Figure S18. ^{13}C NMR spectrum of synthetic (*S*)-violapyrone C in CD_3OD (125 MHz).

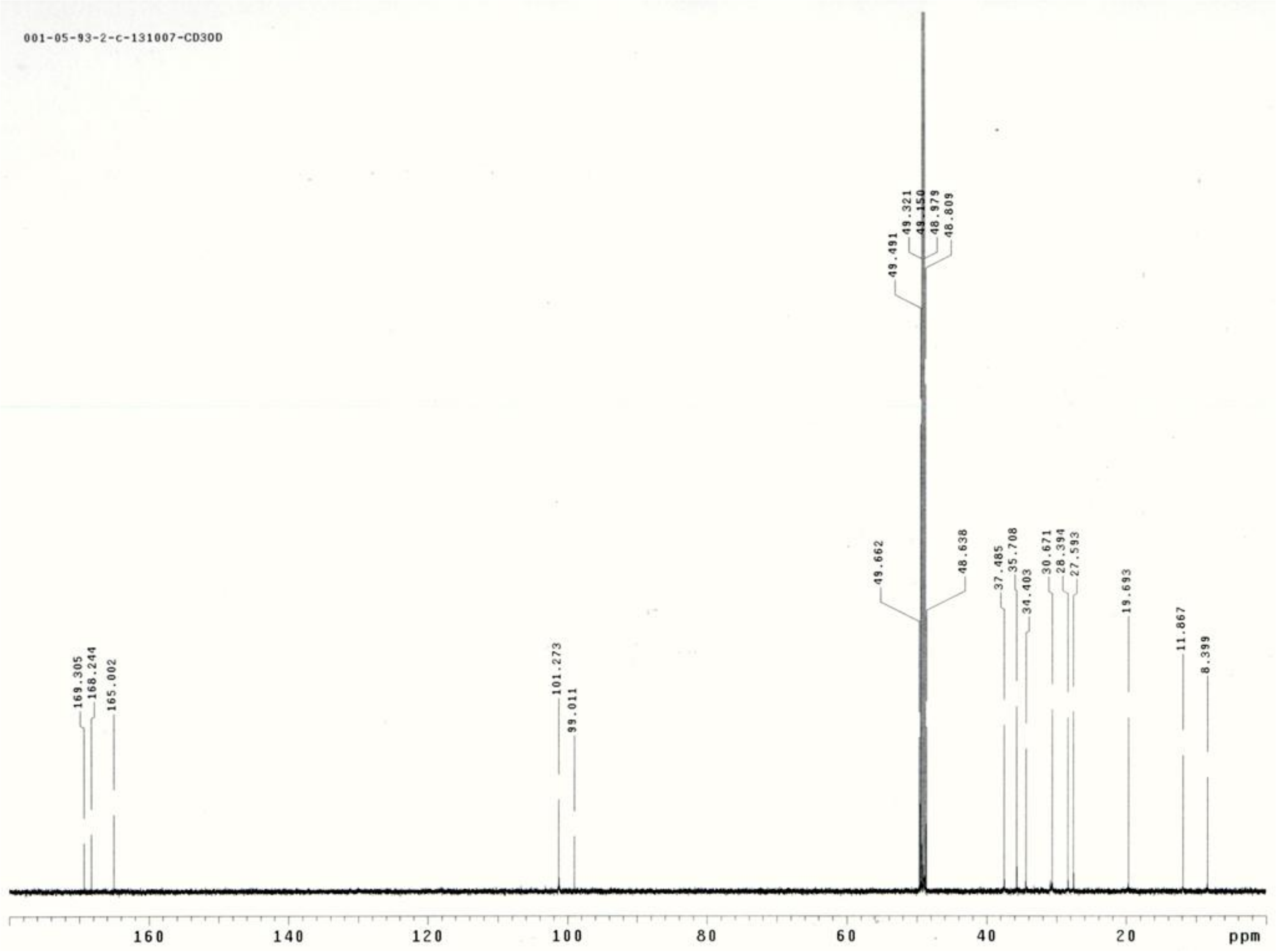

© 2014 by the authors; licensee MDPI, Basel, Switzerland. This article is an open access article distributed under the terms and conditions of the Creative Commons Attribution license (http://creativecommons.org/licenses/by/3.0/).
